# Predictors of severe disease in *Streptococcus dysgalactiae* subsp. *equisimilis* bacteremia: a population-based study

**DOI:** 10.1186/s12879-025-10966-8

**Published:** 2025-04-22

**Authors:** Miia Saukkosaari, Janne Aittoniemi, Reetta Huttunen, Tiina Luukkaala, Sari Rantala

**Affiliations:** 1 Department of Internal Medicine, Tampere University Hospital, Elämänaukio 2, Tampere, 33520 Finland; 2 Faculty of Medicine and Health Technology, Tampere University, Tampere, Finland; 3 Fimlab Laboratories, Tampere, Finland; 4 Research, Development and Innovation Center, Tampere University Hospital, Tampere, Finland; 5 Health Science, Faculty of Social Science, Tampere University, Tampere, Finland

**Keywords:** *Streptococcus dysgalactiae* subspecies *equisimilis*, SDSE, Bacteremia, Risk factor, Mortality, Disease severity

## Abstract

**Background:**

*Streptococcus dysgalactiae* subsp. *equisimilis* (SDSE) is a leading cause of invasive β-hemolytic streptococcal infections in many countries and is increasingly recognized as a cause of severe disease. However, clinical data on severe SDSE disease remain limited. The aim of this study was to identify predictors of severe disease in SDSE bacteremia.

**Methods:**

This retrospective study covered 217 episodes of SDSE bacteremia in 211 adult patients in the Pirkanmaa area, Finland from August 2015 to June 2018. Severe disease was defined as admission to an intensive care unit (ICU) and/or death.

**Results:**

10% of the patients had severe disease, and the overall 30-day case-fatality rate was 7.8%. Alcohol abuse (odds ratio [OR] 5.5 [95% confidence interval (CI) 1.1–28], *p* = 0.038) and malignancy (OR 4.2 [1.3–13], *p* = 0.014) were significantly associated with severe disease. Unconsciousness (OR 23 [1.9–271], *p* = 0.018), dyspnea (OR 5.4 [1.7–17], *p* = 0.005) or falling (OR 3.8 [1.1–13], *p* = 0.031) as the first sign or symptom predicted severe disease.

**Conclusion:**

Alcohol abuse, malignancy, as well as unconsciousness, dyspnea and falling as first signs of infection were associated with severe disease in SDSE bacteremia. These novel findings expand our knowledge of SDSE bacteremia and provide valuable insights for identifying patients at the highest risk of severe disease.

## Background

*Streptococcus dysgalactiae* subsp. *equisimilis* (SDSE) belongs to the group of β-hemolytic streptococci [1], and it has become the leading cause of invasive β-hemolytic streptococcal disease in several countries [2–5]. SDSE is increasingly recognized as a cause of severe invasive infections [6–9]. Up to 70% of SDSE bacteremia patients have shown signs of severe disease in previous studies [8, 10]. The mortality rate of patients with SDSE bacteremia varies from 2 to 20% [7, 8, 11, 12]. The need for intensive care treatment in SDSE bacteremia has been less well studied, and it varies between 7% and 26% [8, 13, 14].

Previous studies of β-hemolytic streptococcal bacteremia (Lancefield group A, B, C or G) have indicated that chronic heart or lung disease and alcohol abuse are associated with higher case-fatality, whereas cellulitis as the site of infection is linked to a more favourable outcome [13–15]. In studies concerning SDSE bacteremia, age over 60 years and bacteremia without a focus have been independently associated with increased case-fatality [10].

The incidence of SDSE bacteremia has been rapidly increasing, but clinical data on severe disease remain limited [9, 10]. We present a three-year population-based retrospective study on predictors of severe SDSE disease. The aim of our study was to identify markers of severe disease, focusing on underlying diseases, first signs and symptoms, clinical manifestations, clinical picture and laboratory test results.

## Methods

The Pirkanmaa health district (HD) is in western Finland and has a population of 532,000 residents. The HD incorporates one tertiary care hospital (Tampere University Hospital), four regional hospitals and over 20 municipal primary/secondary health care units.

The Department of Hospital Hygiene and Infection Control maintains a register of hospital infections and antimicrobial use (the SAI register), which includes every positive blood culture sample in Finland. Fimlab laboratories in Tampere analyse and culture all blood samples from the Pirkanmaa HD.

Blood cultures set up from August 2015 to October 2017 were collected into BacT/Alert Aerobic (FA Plus) and Anaerobic (FN Plus) blood-culture bottles and incubated in an automated microbial detection system (BacT/Alert 3D, bioMérieux, Marcy l’Etoile, France). From November 2017 to July 2018 the blood cultures were collected into BD BACTEC Plus Aerobic/F and Lytic/10 Anaerobic/F culture vials and incubated in a BD BACTEC FX blood-culture system (Becton Dickinson, Sparks, MD, USA). SDSE was primarily determined on the basis of typical large colony-forming growth and β-hemolysis on blood agar plates. Up until February 2017, identification of the bacteria was based on latex agglutination and Lancefield grouping (PathoDxtraTM Strep Grouping Kit, Thermo Scientific, Basingstoke, Hants, UK), plus confirmation using API^®^ 20 STREP equipment (bioMérieux, Marcy l’Etoile, France) or matrix-assisted laser-desorption/ionization time-of-flight mass spectrometry, i.e., MALDI-TOF MS (VITEK^®^ MS, bioMérieux, Marcy l’Etoile, France) [1]. Since March 2017, the MALDI-TOF MS method has been used. MALDI-TOF analysis provided results for S. dysgalactiae subsp. dysgalactiae/equisimilis, which was interpreted as S. dysgalactiae subsp. equisimilis associated with human disease. Antimicrobial susceptibility testing was based on the The European Committee on Antimicrobial Susceptibility Testing (EUCAST) standards [16].

This retrospective, population-based study covers the period from August 2015 to July 2018. The SDSE bacteremia cases were identified from the SAI register, and all adults (over 18 years) treated in Pirkanmaa HD hospitals during the study period were included. Each patient had at least one positive blood culture for SDSE and clinical signs of infection. In total, 230 positive blood cultures were identified. An infectious disease specialist (SR) reviewed and collected data from the electronic medical records. Epidemiological and clinical aspects of the study population have been published earlier [5, 17].

Severe disease was defined as ICU admission and/or death within 30 days of a positive blood culture. Alcohol abuse was defined as a known social or medical problem related to alcohol or alcohol consumption exceeding recommendations (12 or more units per week for women and 23 or more units for men). Malignancy was defined as a history of solid organ or hematological malignancy. Information about alcohol use, history of malignancy, and chronic diseases was obtained from the patients’ medical reports. Immunosuppressive treatment was defined as systemic glucocorticoid therapy, active cytostatic therapy, and/or the use of biological medications. The definition of streptococcal toxic shock syndrome (STSS) included the identification of SDSE in the blood, septic shock and multiorgan failure (MOF). Recurrent bacteremia was defined as a new positive blood culture occurring more than 30 days after the first one.

To assess the association between clinical variables, leukopenia and thrombopenia and severe disease, Fisher’s exact test was used. The results were confirmed through unadjusted and multivariable-adjusted logistic regression analyses and presented as odds ratios (ORs) with 95% confidence intervals (CIs). Age and sex were also included in multivariable-adjusted analyses. Backward-stepwise approach was used for logistic regression analysis. It starts with a full (saturated) model and at each step eliminates the least statistically significant variable from the regression model to find a reduced model that best explains the data. P-values under 0.05 were considered statistically significant. A Bonferroni correction for multiple comparisons was applied. IBM SPSS Statistics for Windows, Version 29 (Armonk, NY: IBM Corp) was used for statistical analysis.

The authors used GPT-4.0, produced by OpenAI, to enhance the language and readability of this manuscript. After using this tool, the authors reviewed and edited the content as needed and take full responsibility for the content of the publication.

## Results

Between August 2015 and July 2018, a total of 230 episodes of SDSE bacteremia were identified within the Pirkanmaa HD region. Clinical patient information was available for 217 bacteremia episodes involving 211 patients. The overall incidence of severe disease was 10%. Within 30 days of a positive blood culture, eight patients (3.7%) were admitted to an ICU and 17 died, resulting in an overall case-fatality rate of 7.8%.

**Table 1:** Underlying disease in severe *Streptococcus dysgalactiae* subsp. *equisimilis* bacteremia from August 2015 to July 2018, Pirkanmaa HD^a^. Unadjusted and multivariable-adjusted logistic regression analysis was performed. All variables listed in the table, were included in multivariable-adjusted model. Results were shown using odds ratios (OR) with 95% confidence intervals (CI)

				Admission to ICU^b^ and/or death					
	All episodes *N* = 217			Severe disease *n* = 22		Unadjusted		Multivariable- adjusted	
	N		(% of episodes)	n	(% of subgroup)	OR	(95% CI)	OR	(95% CI)
Gender									
Male	130		(60)	13	(59)	1.0		1.0	
Female	87		(40)	9	(41)	1.0	(0.4–2.5)	1.3	(0.4–4.1)
Age, years									
18–49	19		(8.8)	2	(9.1)	1.0		1.0	
50–64	31		(14)	2	(9.1)	0.6	(0.1–4.6)	0.3	(0.03–3.8)
65–79	83		(38)	10	(45)	1.2	(0.2–5.8)	0.5	(0.1–4.1)
≥ 80	84		(39)	8	(36)	0.9	(0.2–4.6)	0.3	(0.03–3.5)
Obesity^c^	98		(45)	8	(36)	0.7	(0.3-2.0)	1.1	(0.3–3.6)
Alcohol abuse	22		(10)	6	(27)	4.2	(1.4–12)	5.5	(1.1–28)
Malignancy	62		(29)	10	(45)	2.3	(0.9–5.6)	4.2	(1.3–13)
Cardiovascular diseases	97		(45)	14	(64)	2.4	(0.9–5.9)	1.4	(0.2–12)
Coronary artery disease	53		(24)	9	(41)	2.4	(1.0-5.9)	1.7	(0.4–7.4)
Chronic heart failure	74		(34)	11	(50)	2.1	(0.9–5.1)	2.2	(0.3–15)
Diabetes	81		(37)	10	(45)	1.5	(0.6–3.5)	1.5	(0.5–4.7)
Rheumatic disease	14		(6.5)	2	(9.1)	1.5	(0.3–7.3)	2.5	(0.3–22)
Chronic lung disease	37		(17)	6	(27)	2.0	(0.7–5.5)	1.6	(0.1-4)
Asthma	20		(9.2)	2	(9.1)	1.0	(0.2–4.6)	0.5	(0.02-13)
COPD	14		(6.5)	4	(18)	4.1	(1.2–14)	2.5	(0.1–57)
Chronic kidney disease	30		(14)	1	(4.5)	0.3	(0.04–2.1)	0.2	(0.02–1.6)
Chronic liver disease	10		(4.6)	2	(9.1)	2.3	(0.5–12)	0.7	(0.1–8.5)
Neurological disease	44		(20)	4	(18)	0.9	(0.3–2.7)	1.2	(0.3-5.0)
Immunosuppressive treatment^d^	28		(13)	2	(9.1)	0.7	(0.1–2.9)	0.4	(0.1–3.1)

^a^Health district

^b^Intensive care unit

^c^Body mass index > 30 kg/m^2^

^d^Systemic glucocorticoid treatment, active cytostatic therapy, and/or the use of biological medications

The demographic and clinical characteristics of patients with SDSE bacteremia are detailed in Table 1. Most patients were male and over 65 years of age. Age was not associated with severe disease. Alcohol abuse (OR 5.5 [95% CI 1.1–28], *p* = 0.038), and malignancy (OR 4.2 [1.3–13], *p* = 0.014) as an underlying disease were associated with severe disease in multivariable-adjusted logistic regression analysis when risk factors were entered simultaneously into the model. In backward-stepwise logistic regression analysis, chronic heart failure (OR 2.9 [95% CI 1.1–7.9], *p* = 0.037) and chronic obstructive pulmonary disease (COPD) (OR 4.7 [1.0–21], *p* = 0.043), in addition to alcohol abuse and malignancy, were associated with severe disease. Persons with HIV and those who injected drugs were absent in this study.

**Fig. 1:**
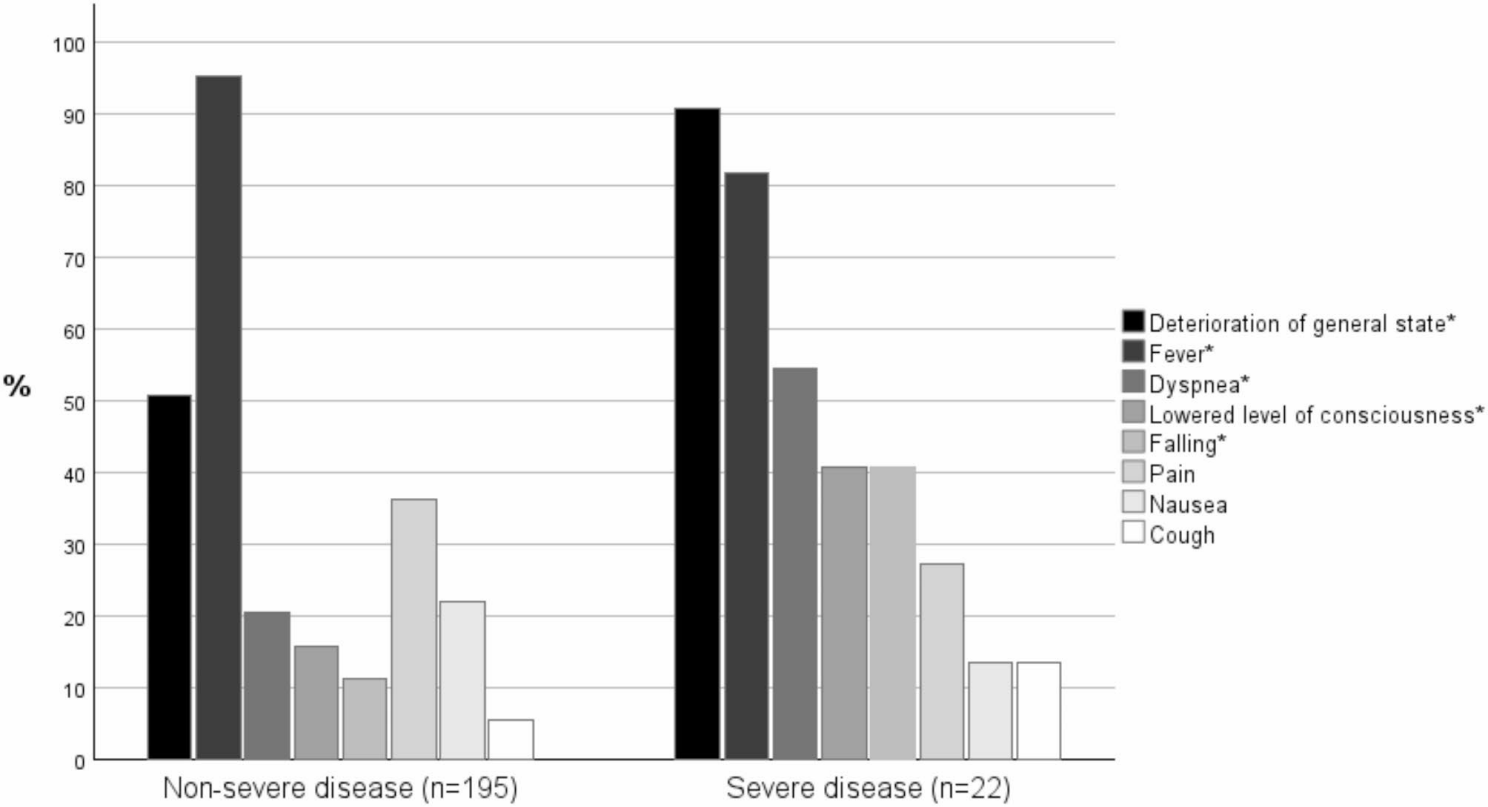
First signs and symptoms of non-severe and severe *Streptococcus dysgalactiae* subsp. *equisimilis* bacteremia in the Pirkanmaa health district, Finland, during the study period of August 2015 to July 2018. *Statistical significance (*p* < 0.05) was found between the groups in unadjusted analysis.

Patients in the severe disease group showed more frequent first signs and symptoms than those in the non-severe disease group (Fig. 1). Comparison of the first signs and symptoms in the severe and non-severe group is shown in Table 2. Unconsciousness (OR 23 [95% CI 1.9–271], *p* = 0.018), dyspnea (OR 5.4 [1.7–17], *p* = 0.005) and falling (OR 3.8 [1.1–13], *p* = 0.031) as first signs and symptoms were significantly associated with severe disease in multivariable-adjusted models.

**Table 2:** First signs and symptoms of non-severe and severe *Streptococcus dysgalactiae* subsp. *equisimilis* bacteremia during the study period from August 2015 to July 2018, Pirkanmaa HD^a^ (*N* = 217). Unadjusted and multivariable-adjusted logistic regression analyses were performed. All variables listed in the table, were included in multivariable-adjusted model. Age and gender were included as covariates in multivariable-adjusted model. Results were shown using odds ratios (OR) with 95% confidence intervals (CI)

			Admission to ICU^b^ and/or death					
	Non-severe disease *n* = 195		Severe disease *n* = 22		Unadjusted		Multivariable-adjusted	
	n	(% of patients)	n	(%)	OR	(95% CI)	OR	(95% CI)
Level of consciousness								
Normal	164	(84)	13	(59)	1.0		1.0	
Confusion	30	(15)	6	(27)	2.5	(0.9–7.2)	2.4	(0.7–8.2)
Unconsciousness	1	(0.5)	3	(14)	38	(3.7–390)	23	(1.9–271)
Fever								
No	9	(4.6)	4	(18)	1.0		1.0	
Yes	186	(95)	18	(82)	0.2	(0.06–0.8)	0.2	(0.03-1.0)
Nausea								
No	152	(78)	19	(86)	1.0		1.0	
Yes	43	(22)	3	(14)	0.6	(0.2-2.0)	0.7	(0.1-3.0)
Falling								
No	173	(89)	13	(59)	1.0		1.0	
Yes	22	(11)	9	(41)	5.4	(2.1–14)	3.8	(1.1–13)
Dyspnea								
No	155	(79)	10	(45)	1.0		1.0	
Yes	40	(21)	12	(55)	4.7	(1.9–12)	5.4	(1.7–17)
Cough								
No	184	(94)	19	(86)	1.0		1.0	
Yes	11	(5.6)	3	(14)	2.6	(0.7–10)	2.6	(0.5–14)
Pain								
No	124	(64)	16	(73)	1.0		1.0	
Yes	71	(36)	6	(27)	0.7	(0.2–1.7)	0.9	(0.3–3.3)
Deterioration of general state								
No	96	(49)	2	(9.1)	1.0		1.0	
Yes	99	(51)	20	(91)	9.7	(2.2–43)	4.6	(0.9–24)

^a^Health district

^b^Intensive care unit

The distribution of clinical manifestations is shown in Table 3. Of the 149 cellulitis cases, 4.7% were in the severe disease group. Cellulitis was associated with non-severe disease. The significance persisted even after applying the Bonferroni correction. One case of necrotizing fasciitis (NF) (0.5% prevalence) occurred in the severe disease group and three cases of infective endocarditis (IE) (1.4% prevalence) occurred in the non-severe disease group. The prevalence of STSS was 4.6%. There was no difference between severe and non-severe disease when the focus was unknown.

**Table 3:** Presenting clinical manifestations in non-severe and severe *Streptococcus dysgalactiae* subsp. *equisimilis* bacteremia from August 2015 to July 2018, Pirkanmaa HD^a^ (*N* = 217). One patient may have one or more clinical manifestations

			Admission to ICU^b^ and/or death		
	Non-severe disease *n* = 195		Severe disease *n* = 22		
	n	(%)	n	(%)	p-value
Purulent skin infection	38	(20)	5	(23)	0.778
Cellulitis	142	(73)	7	(32)	< 0.001
Necrotizing fasciitis	0		1	(4.5)	
STSS^c^	1	(0.5)	9	(41)	< 0.001
Bursitis	3	(1.5)	0		
Abscess	12	(6.2)	1	(4.5)	1.000
Pneumonia	29	(15)	6	(27)	0.136
Empyema	2	(1.0)	0		
Osteomyelitis all	2	(1.0)	1	(4.5)	0.275
Spondylitis	1	(0.5)	1	(4.5)	0.193
Arthritis all	6	(3.1)	2	(9.1)	0.189
Periprosthetic joint infection	4	(2.1)	0		
Endocarditis	3	(1.5)	0		
Intra-abdominal infection	2	(1.0)	1	(4.5)	0.275
Puerperal sepsis	4	(2.1)	0		
Foreign body infection	3	(1.5)	0		
Endophthalmitis	1	(0.5)	0		
Aortitis	0		1	(4.5)	
Focus unknown	24	(12)	5	(23)	0.186

^a^Health district

^b^Intensive care unit

^c^Streptococcal toxic shock syndrome

None of the three cases of IE required ICU admission. One of these patients underwent a surgical procedure involving a biological prosthesis in the aortic position, and two patients (67%) experienced arterial thrombosis.

Eight patients were treated in an ICU. Of these, one had arthritis, three had a skin infection, two had both a skin infection and pneumonia, one had a skin infection along with an abscess, arthritis, and osteomyelitis, and one had an unknown infection focus.

**Table 4:** Clinical characteristics of *Streptococcus dysgalactiae* subsp. *equisimilis* bacteremia during the study period from August 2015 to July 2018, Pirkanmaa HD (*N* = 217). Unadjusted and multivariable-adjusted logistic regression analyses were performed, expressing results by odd ratio (OR) with 95% confidence intervals (CI). All variables listed in the table, if applicable, were included in multivariable-adjusted model. Age and gender were included as covariates in multivariable-adjusted model

	Survivors *n* = 200		Non-survivors *n* = 17		Unadjusted		Multivariable-adjusted	
	n	(%)	n	(%)	OR	(95% CI)	OR	(95% CI)
Septic shock^a^	7	(3.5)	10	(59)	39	(12–134)	40	(5.2–307)
Needed mechanical ventilation^b^	1	(0.5)	1	(5.9)	12	(0.7–208)	1.6	(0.02–153)
Needed hemodialysis^c^	1	(0.5)	0			NA		NA
Lowered level of consciousness^d^	41	(21)	12	(71)	9.3	(3.1–28)	6.9	(1.4–33)
DIC^e^	10	(5.0)	0		0.9	(0.9-1.0)		NA
Multiorgan failure	5	(2.5)	7	(41)	27	(7.3–101)	0.9	(0.1–15)
Thromboembolic complication^f^	10	(5.0)	2	(12)	2.7	(0.5–14)	1.1	(0.1–14)
Underwent surgical intervention	32	(16)	5	(29)	2.2	(0.7–6.6)	1.0	(0.2–6.3)

^a^ Use of vasopressor and lactate level > 2 mmol/L

^b^ Needed mechanical ventilation within 30 days of positive blood culture

^c^ Additionally 3 patients in non-severe group were in hemodialysis prior bacteremia

^d^ Unconsciousness or confusion at least once during the first two days after positive blood culture

^e^ Disseminated intravascular coagulation; thrombocytes lower than 100 × 10^9^ / l

^f^ Venous or arterial event

HD = Health district

NA = Not applicable

The clinical characteristics of survivors and non-survivors is presented in Table 4. Septic shock and a decreased level of consciousness during the first two days of hospitalization were more common among non-survivors (*p* < 0.001 and *p* = 0.017). Multiorgan failure (*p* < 0.001) was more frequently observed in the non-survivor group as well, but only in the unadjusted statistical model. There was no difference between the groups in the need for surgical procedures. The most often required procedures were resections or amputations of the lower limb, accounting for 22% (8/37) of all operations. Four patients (11%) required revision or debridement of their knee prostheses, four patients underwent exploratory incision, and four underwent lower-limb vascular surgical procedures. An abdominal procedure or surgery was also carried out in four cases.

An elevated level of C-reactive protein (CRP; above the overall median of 116 mg/L), high leukocyte counts (above the overall median of 14.3 × 10^9^/L) or increased creatinine levels (above 120 µmol/L) at hospital admission were not associated with a higher risk of severe disease. Seven patients had leukopenia (white blood cell count < 4.0 × 10^9^/L) on admission to hospital, and two of these (29%) were in the severe disease group. Twelve patients had thrombocytopenia (platelet count < 100 × 10^9^/L) on admission, and two of these (17%) were in the severe disease group.

In the non-severe group, cefuroxime was the initial antibiotic treatment for 74% of the patients, compared with 38% in the severe disease group (OR 0.2 [95% CI 0.1–0.5], *p* < 0.001). Conversely, ceftriaxone was initiated in 38% of patients in the severe disease group and 12% in the non-severe group (OR 4.3 [1.6–11], *p* = 0.003). Piperacillin-tazobactam was the third most common antibiotic in the severe disease group, accounting for 14% of cases, compared with 3.6% in the non-severe group (OR 4.2 [1.0–18], *p* = 0.048). None of the patients in the severe disease group received benzylpenicillin as the initial antibiotic, while only 2.6% of those in the non-severe group did.

The initial antibiotic treatment was microbiologically effective in all patients. After two days, the treatment was narrowed to benzylpenicillin in 58 patients (30%) within the non-severe group and three patients (14%) in the severe disease group. Throughout the infection, 52 patients (24%) received clindamycin (24% in severe disease group and 25% in non-severe disease group), and its use was not associated with disease severity (OR 1.0 [95% CI 0.3–2.8]). Immunoglobulin treatment was not used. All strains were susceptible to penicillin and cephalosporins. Antimicrobial resistance rates were 1.8% for clindamycin and 10% for erythromycin.

Six male patients experienced two episodes of SDSE bacteremia. The median age was 71 years, and five of the six patients were at least 65 years old. The patients had the following underlying conditions: one had diabetes, one cardiovascular disease, one pulmonary disease, one had malignancy, one both cardiovascular disease and diabetes, and one had cardiovascular disease, alcohol abuse and liver disease. In each episode, a skin infection was the primary clinical manifestation. During the second episode, one of the six patients experienced severe disease.

## Discussion

This study highlights the new findings that alcohol abuse and malignancy are predictors of severe disease in SDSE bacteremia. Alcoholism, chronic heart or lung disease and immune incompetence have been previously reported as being predisposing factors of case fatality in a few studies on β-hemolytic streptococcal bacteremia [13, 14], but not specifically in SDSE bacteremia studies. Chronic alcohol use has been shown to both predispose individuals to sepsis and worsen its outcomes [18]. Additionally, previous studies have shown that having any type of cancer increases mortality in sepsis patients [19]. Our analysis also revealed that chronic heart failure and COPD were associated with severe disease, which is a new finding in SDSE bacteremia. Septic infection can worsen heart failure symptoms, and heart failure has been associated with significantly higher in-hospital mortality rates in sepsis patients [20]. In the present study we found no significant association between age and severe disease. Hanada et al. recently reported age ≥ 60 years to be associated with case-fatality [10].

This study adds to our knowledge that unconsciousness, dyspnea and falling as first signs and symptoms are clinical indicators of severe disease in SDSE bacteremia. An altered level of consciousness, and dyspnea have been previously associated with mortality in β-hemolytic streptococcal bacteremia [14], but they have not been studied in SDSE bacteremia. These conditions are significant symptoms of septic infection, and they are also among the criteria of quick Sequential (Sepsis-related) Organ Failure Assessment (qSOFA) [21]. Given this information, patients exhibiting these symptoms should prompt fast clinical decisions.

We confirmed previous findings showing that cellulitis is associated with non-severe disease in SDSE bacteremia [10]. The prevalence of NF in the present study was 0.5%, notably lower than the rates in previous studies, ranging from 1.0 to 18% [4, 11, 22]. The prevalence of IE was 1.4%, which is also lower than in earlier studies, in which reported rates have ranged from 1.7 to 6% [4, 22, 23]. SDSE-caused IE has been reported to be associated with a rapid onset of symptoms and a high mortality rate [23–25]. None of the IE patients in this study required ICU admission or died. However, two of the three patients experienced arterial thrombotic events, and one underwent valvular replacement. A possible explanation for the lower rates of NF and IE observed in this study is its population-based design. It includes not only a tertiary hospital but also several primary health care units, which encompass a broader range of bacteremia cases, including milder forms. The results of a nationwide study in Japan [10] were similar to ours, strengthening this finding.

In previous SDSE bacteremia studies up to 70% of patients have shown signs of severe disease and 7–26% have been admitted to ICUs [8, 10, 14, 26]. The prevalence of severe disease (10%) and the need for ICU treatment (3.7%) were notably lower in the present study. There are likely several reasons for the result. Some patients were treated in monitoring wards that support treatments such as non-invasive ventilation and vasopressor administration, but which are not classified as intensive care units. Additionally, few patients were excluded from ICU care as they were not expected to benefit from intensive treatment. The difference may also be explained by the population-based design. The definition of severe disease varies between studies, which can also affect the results. Virulence factors may also have a role [8]. However, the mortality rate (7.8%) is entirely consistent with those in other reports of SDSE bacteremia [6–8, 27].

In the present study, patients with severe disease were significantly more likely to be empirically treated with broad-spectrum antibiotics, such as ceftriaxone or piperacillin-tazobactam. We found that clindamycin treatment was not associated with the severity of the disease or its outcome. The use of clindamycin has previously been associated with reduced mortality in severe *Streptococcus pyogenes* infections, but the same has not been shown in non-A, non-B infections [28]. After the blood-culture results were available, antibiotic treatment was narrowed to penicillin in 30% of cases in the non-severe disease group and in 14% in the severe disease group. In a study on complicated skin infections, patients treated with broad-spectrum antibiotics had higher failure rates compared with those treated with penicillin [29]. This highlights the importance of de-escalating antibiotics once the results of blood culture are defined.

In this study, clindamycin resistance was low (1.8%), which is in line with reports from Norway and Denmark [12, 30]. However, in other regions, clindamycin resistance has varied between 4% and 34.6% [4, 6, 31]. Erythromycin resistance has ranged from 5 to 42.3% in different studies [4, 6, 12, 29, 31]. In our study we found an erythromycin resistance rate of 10%, which is in line with rates reported in other Nordic countries [12, 30].

Data concerning the association between high CRP levels and mortality are conflicting [10, 14, 22]. In this study, high CRP levels were not associated with severe disease. We found no association between leukopenia, thrombocytopenia, or elevated serum creatinine levels and severe disease, despite previous studies linking these factors to higher case-fatality rates and post-infective sequelae in SDSE bacteremia [10, 22].

The strengths of this study include its population-based design, the reliable collection of patient data by a single infectious disease specialist (SR), and a sufficiently large study population. There are also limitations. The retrospective study design limits the ability to use clinical scores, which could have facilitated a comparison of our results with previous studies.

## Conclusion

Alcohol abuse and malignancy were linked to severe disease in SDSE bacteremia. Among the initial signs and symptoms, unconsciousness, dyspnea, and falling were indicative of severe disease. These findings can be utilized in frontline clinical practice to identify patients at the highest risk of severe disease and mortality.

## Data Availability

The datasets generated and/or analyzed during this study are not publicly available due to the protection of participant confidentiality. For inquiries regarding the datasets or requests for additional analyses, please contact the corresponding author.

## Abbreviations

CI: Confidence interval
COPD: Chronic obstructive pulmonary disease
CRP: C-reactive protein
HD: Health district
ICU: Intensive care unit
IE: Infective endocarditis
MOF: Multiorgan failure
NF: Necrotizing fasciitis
qSOFA: Quick Sequential (Sepsis-related) Organ Failure Assessment
SDSE: Streptococcus dysgalactiae subspecies equisimilis
STSS: Streptococcal toxic shock syndrome
